# All roads lead to Rome: the plasticity of gut microbiome drives the extensive adaptation of the Yarkand toad-headed agama (*Phrynocephalus axillaris*) to different altitudes

**DOI:** 10.3389/fmicb.2024.1501684

**Published:** 2025-01-08

**Authors:** Jianghao Du, Peng Zheng, Weizhen Gao, Qianru Liang, Lin Leng, Lei Shi

**Affiliations:** Xinjiang Key Laboratory for Ecological Adaptation and Evolution of Extreme Environment Biology, College of Life Sciences, Xinjiang Agricultural University, Ürümqi, China

**Keywords:** *Phrynocephalus axillaris*, altitude gradients, gut microbiome, 16S rRNA, LC-MS metabolomics, plasticity

## Abstract

The gut microbiome was involved in a variety of physiological processes and played a key role in host environmental adaptation. However, the mechanisms of their response to altitudinal environmental changes remain unclear. In this study, we used 16S rRNA sequencing and LC-MS metabolomics to investigate the changes in the gut microbiome and metabolism of the Yarkand toad-headed agama (*Phrynocephalus axillaris*) at different altitudes (−80 m to 2000 m). The results demonstrated that Firmicutes, Bacteroidetes, and Proteobacteria were the dominant phylum, Lachnospiraceae and Oscillospiraceae were the most abundant family, and the low-altitude populations had higher richness than high-altitude populations; Akkermansiaceae appeared to be enriched in high-altitude populations and the relative abundance tended to increase with altitude. The gut microbiome of three populations of *P. axillaris* at different altitudes was clustered into two different enterotypes, low-altitude populations and high-altitude populations shared an enterotype dominated by *Akkermansia*, *Kineothrix*, *Phocaeicola*; intermediate-altitude populations had an enterotype dominated by *Mesorhizobium*, *Bradyrhizobium*. Metabolites involved in amino acid and lipid metabolism differed significantly at different altitudes. The above results suggest that gut microbiome plasticity drives the extensive adaptation of *P. axillaris* to multi-stress caused by different altitudes. With global warming, recognizing the adaptive capacity of wide-ranging species to altitude can help plan future conservation strategies.

## 1 Introduction

The impact of global climate change on biodiversity is a focus of concern due to the rapid loss of biodiversity (Ceballos et al., 2017). Revealing the long-term adaptive and phenotypic plasticity of organisms to environmental change is extremely challenging due to the interaction of multiple biotic and abiotic factors that generate complex biological responses (Serén et al., 2023). Understanding plasticity responses to environmental pressures focuses on widely distributed species, especially with extensive altitudinal gradients. Altitude is a complex ecological factor that encompasses a wide range of potential stressors, (temperature, oxygen pressure, and ultraviolet radiation), it has a profound effect on the phenotype, genotype, and geographic distribution of animals. Altitudinal gradients serve as powerful “natural experiments” for testing ecological and evolutionary adaptations (Körner, 2007). Plasticity buffers environmental changes along altitudinal gradients and ultimately evolves specific strategies in genetics, physiology, morphology, and behavior to adapt to local environments (Beldade et al., 2011; Enriquez-Urzelai et al., 2020; Grether, 2005). Ectotherms are more vulnerable to environmental changes than endotherms due to their strong dependence on external conditions for regulating body temperature (Paaijmans et al., 2013). However, several studies have demonstrated that ectotherms exhibit remarkable adaptability to varying altitudes, with significant plasticity in response to environmental changes, including gene expression, metabolic rate regulation, growth, and reproductive strategies (Niu et al., 2022; Yang et al., 2019; Zhang et al., 2023). The changes in gut microbiome at different altitudes and their adaptive significance remain poorly studied (Chen et al., 2022).

The gut microbiome plays an important role in various animal physiological activities, including health, growth, development, and behavior. It can even influence the nervous system through the secretion of metabolites (Ling et al., 2018; Maritan et al., 2024; Pascale et al., 2018; Strati et al., 2017; Sun et al., 2024). Consequently, the host and its gut microbiome coexist in a dynamic mutualistic relationship (Karl et al., 2018). Understanding the origins of the gut microbiome is critical for the identification and interpretation of potential fitness-related traits for the host. The presence of recurrent microbial compositional patterns in the gut microbiome is characterized by differences in the abundance of signature taxa, referred to as enterotypes (Wang et al., 2014). Enterotype was first reported in humans and later extended to other mammalian hosts (Arumugam et al., 2011; Costea et al., 2018; Couch et al., 2021; Hicks et al., 2018). That enterotype can serve as a valuable tool for studying gut microbiome from different habitats and taxa and their effects on the host. On the other hand, metabolomics analyses provide opportunities to assess metabolic regulatory mechanisms and discover new biomarkers of animal responses to environmental stresses (Lankadurai et al., 2013). Fecal metabolomics analysis can identify most of the metabolites present in a sample that reflects the net result of nutrient ingestion, digestion, and absorption by both the gut microbiome and the gut (Karu et al., 2018). Therefore, fecal metabolomics is widely considered a key tool for studying the relationship between hosts and their gut microbiome (Jonas et al., 2018).

Reptiles are highly diverse ectothermic vertebrates, of which the Sauria (lizards) contain 7458 species.^1^ In recent years, the studies of the gut microbiome in lizards have attracted considerable attention. Many factors can affect the gut microbiome of lizards, as demonstrated by studies of several species including (*Shinisaurus crocodilurus*), *Tak*ydro*mus septentrionalis*, *Japalura sensu lato*, *Teratoscincus roborowskii*, *Sceloporus grammicus*, *Eremias multiocellata*, and *Phrynocephalus vlangalii*. These factors include captive conditions (Tang et al., 2020; Zhou et al., 2020), gut and oral compartments (Tian et al., 2022), temperature changes (Yang et al., 2024), diet (Gao et al., 2023; Montoya-Ciriaco et al., 2020), urbanization (Littleford-Colquhoun et al., 2019), and altitudes (Zhang et al., 2018).

*Phrynocephalus axillaris* is a representative and dominant species in the Northwest Desert Region in China, with prominent desert adaptive ability and a wide altitudinal distribution from Aiding Lake in the Turpan Basin, which is 155 m below sea level, to the Altun-Kunlun Mountains, which is 3,000 m above sea level (Zhao and Zhao, 1999), and the altitudinal gradient of more than 3000 m means that the species needs to cope with different ecological environments. So it is an ideal mode to reveal the impact of altitude changes on gut microbiome. Located in eastern Xinjiang, the Turpan Basin is the second deepest lowland in the world (Fang et al., 2010). And it is a typical arid inland basin characterized by an extremely dry environment, low average annual precipitation, low relative atmospheric humidity, water scarcity and fragile ecosystems, which has resulted in a desert plant community characterized by more *Poaceae* species and annual herbaceous plants (Tu et al., 2024; Domrös et al., 1992). Altun-Kunlun Mountain is located northward toward the Taklamakan Desert, with an arid climate, low annual precipitation, and an alpine steppe, mainly in a desert environment (Chen and Li, 2023). Current studies on *P. axillaris* have involved age and growth (Ran et al., 2023), genetic diversity analysis (Xie et al., 2023), phylogeographic patterns, mitochondrial genome (Chen et al., 2019; Zhang et al., 2010), and sexual dimorphism (Cao et al., 2020). However, the effects of different altitude habitats on the gut microbiome of *P. axillaris* and their ecological adaptation mechanisms are not clear.

Therefore, we used 16S rRNA sequencing and LC-MS metabolomics to investigate the altitude-adaption of gut microbiome and metabolites in widely distributed *P. axillaris*. We aim to understand the co-evolution of the lizard and its gut microbiome, and thus reveal its complex environmental adaptation mechanisms. Our speculations are as follows: (i) Dominant gut microbiome remains relatively stable, and signature taxa show plasticity to respond to different altitudes. (ii) Low temperatures, hypoxia, and strong UV are present at high altitude, leading to an increase in the abundance of probiotics that enhance energy metabolism; and extremely high temperatures at low altitude lead to an enrichment of probiotics that are associated with thermo-tolerance and anti-inflammatory effects. (iii) Decreased fitness of populations at low and high altitude resulted in a similar enterotype associated with stress response, which is different from that of intermediate altitude populations.

## 2 Materials and methods

### 2.1 Fecal sample collection and processing

Individuals of *P. axillaris* were captured from Turpan City, Yuli County, Qiemo County between June 2023 and August 2023, which are positioned at low altitudes (LA, <500 m, *N* = 9, −44 m, −25 m, 369 m), intermediate altitudes (IA, 500–1500 m, *N* = 5, 861 m, 886 m, 1109 m), and high altitudes (HA, >1500 m, *N* = 8, 1892 m) (Supplementary Appendix Table 1). After capture, *P. axillaris* were recorded for sex and weight. Subsequently, the lizards were individually maintained in 21 × 14 × 13 cm (L × W × H) plastic boxes, and fecal sampling was used to check for excretion every 1 h to ensure that fresh fecal samples were collected. Fecal samples were collected under aseptic conditions using sterile tweezers and completed fresh fecal samples were collected into sterile cryopreservation tubes, labeled, and stored in liquid nitrogen (Gao et al., 2023). Samples of feces should be collected without providing food to guarantee that they accurately represent the gut microbiota in the field. We collected 22 fecal pellets from 9 samples in LA, 15 fecal pellets from 5 samples in IA, and 32 fecal pellets from 8 samples in HA. After the experiment, all the animals were in good physiological condition and released at the original collection site, and the study was approved by the Animal Welfare and Ethics Committee of Xinjiang Agricultural University (2023014).

### 2.2 DNA extraction sequencing

Microbial DNA was extracted from fecal samples of individuals from different altitude groups by referring to the E.Z.N.A.^®^ Soil DNA Kit (Omega Bio-Tek, Norcross, GA, U.S.) according to the manufacturer’s protocols. To test the quality of the DNA, agarose gel electrophoresis was conducted. The 16S rRNA gene was amplified using the forward primer 27F (5′-AGRGTTYGATYMTGGCTCAG-3′) and the reverse primer 1492R (5′-RGYTACCTTGTTACGACTT-3′) (Callahan et al., 2019). The PCR amplification was performed in a total volume of 20 μL, including: 4 μL 5× FastPfu Buffer, 2 μL 2.5 mM dNTPs, 0.8 μL of each primer (5 μM), 0.4 μL FastPfu Polymerase, and 10ng template DNA. The reactions were performed on GeneAmp^®^9700 with the following amplification conditions: initial denaturation at 95°C for 5 min; followed by 27 cycles of denaturation at 95°C for 30 s, annealing at 55°C for 30 s, elongation at 72°C for 45 s; and final extension at 72°C for 10 min. Amplicons were extracted from 2% agarose gels and purified using the AxyPrep DNA Gel Extraction Kit (Axygen Biosciences, Union City, CA, U.S.) according to the manufacturer’s instructions.

SMRTbell libraries were prepared from the amplified DNA by blunt ligation according to the manufacturer’s instructions (Pacific Biosciences). Purified SMRTbell libraries from the Zymo and HMP mock communities were sequenced on dedicated PacBio Sequel II 8M cells using the Sequencing Kit 2.0 chemistry. Purified SMRTbell libraries from the pooled and barcoded samples were sequenced on a single PacBio Sequel II cell.

### 2.3 Data processing and analysis

PacBio raw reads were processed using the SMRT Link Analysis software version 9.0 to obtain demultiplexed circular consensus sequence (CCS) reads. Raw reads were processed through SMRT Portal to filter sequences for length and quality. OTUs were clustered according to a 98.65% similarity threshold using UPARSE (version 7.1),^2^ and chimeric sequences were identified and removed using UCHIME. The phylogenetic affiliation of each 16S rRNA gene sequence was analyzed by RDP Classifier^3^ against the silva (SSU132) 16S rRNA database using a confidence threshold of 70% (Uparse, 2013). Species taxonomic annotation was performed for each sequence using RDP Classifier (see text footnote 3, version 2.2), chimera sequences were removed with the UCHIME algorithm. These effective tags were clustered into operational taxonomic units (OTUs) based on a sequence similarity threshold of 97% using UPARSE (version 7.0) (Amato et al., 2013; Wang et al., 2007).

The rarefaction analysis is based on Mothur verson.1.30.1 (Schloss et al., 2011) was conducted to reveal the diversity indices, including the Chao1, ACE, Shannon and Simpson diversity indices. The beta diversity analysis was performed using Bray-Curtis to compare the results of the principal component analysis (PCoA) using the community ecology package, R-forge (Vegan 2.0 package was used to generate a PCoA figure) (Dixon, 2003). Adonis to assess significant differences in microbiological structural differences across groups (Kelly et al., 2015), One-way analysis of variance (ANOVA) tests were performed to assess the statistically significant difference of diversity indices among samples. Relative abundance of gut microbiome at different taxonomic levels was tested using the Kruskal-Wallis H test. Differences were considered significant at *P* < 0.05. For identification of biomarkers for highly dimensional colonic bacteria, LEfSe (linear discriminant analysis effect size) analysis was done (Segata et al., 2011), and followed by LDA > 3.5, *P* < 0.05 analysis to screen for differential. The enterotypes were obtained from the relative abundance profiles at the genus level using Jensen-Shannon divergence (JSD) and partitioning around medoid (PAM) clustering in R (version R-3.4.3). JSD is based on the abundance method and is suitable for revealing variations in abundance taxa. To identify genus taxa contributing to enterotype, we applied the SIMPER method (Guo et al., 2021; Lee et al., 2020). Phylogenetic Investigation of Communities by Reconstruction of Unobserved States (PICRUSt2)^4^ program based on the Kyoto Encyclopedia of Genes and Genomes (KEGG) database was used to predict the functional alteration of the microbiome in different samples (Douglas et al., 2019). Significant differences in functional pathways were assessed using one-way ANOVA or Kruskal-Wallis H tests, *P* < 0.05.

### 2.4 LC-MS metabolomics detection and analysis

Fecal samples (100 mg) were individually ground with liquid nitrogen, and the homogenate was resuspended with prechilled 80% methanol and 0.1% formic acid by well vortex, incubated at 4°C for 5 min and centrifuged at 15000 rpm for 5 min. Some of supernatant was diluted to final concentration containing 53% methanol by LC-MS grade water. The samples were transferred to a fresh Eppendorf tube and then were centrifuged at 15000 g, 4°C for 10 min. Finally, the supernatant was injected into the LC-MS/MS system analysis (Want et al., 2006). Liquid sample (100 μL) and prechilled methanol (400 μL) were mixed by well vortexing (Barri and Dragsted, 2013). Cell samples and 4 times prechilled 80% methanol were mixed by well vortexing and then sonicated for 6 min. Repeating this step once again and then operating the same steps as above (Yuan et al., 2012). UHPLC-MS/MS analyses were performed using a Vanquish UHPLC system (Thermo Fisher, Germany) coupled with an Orbitrap Q Exactive™ HF mass spectrometer (Thermo Fisher, Germany).

### 2.5 LC-MS metabolomics data processing and analysis

The raw data files generated by UHPLC-MS/MS were processed using Compound Discoverer 3.1 (CD3.1, Thermo Fisher) to perform peak alignment, peak picking, and quantitation for each metabolite. Peaks with a signal-to-noise ratio (S/N) greater than 50 were considered and normalized to the intensity of the total spectral intensity. After that, peak intensities were normalized to the total spectral intensity. The normalized data was used to predict the formula based on additive ions, molecular ion peaks, and fragment ions. And then peaks were matched with the mzCloud,^5^ mzVault, and MassList databases to obtain accurate and relative quantitative results (Sapozhnikova and Nuñez, 2022). Statistical analyses were performed with the statistical software R (version R-3.4.3).

The KEGG database^6^ was used to annotate these metabolites for annotation (Gil de la Fuente et al., 2017). This was followed by Principal Component Analysis (PCA), Partial Least Squares Regression Analysis (PLS-DA) and Orthogonal Partial Least Squares Discriminant Analysis (OPLS-DA) to visualize the samples for clustering (Szymanska et al., 2011). We used one-way analysis of variance (*t*-test) to calculate statistical significance (*P-*value). VIP > 1 (VIP: projected variable importance), *P-*value < 0.05 and FC > 1 (FC: fold change) were considered as differential metabolites. Based on the log2FC, *P*-value of metabolites, volcano plots and bi-directional bar charts were used to screen for significantly different metabolites (Ahluwalia et al., 2021; Li et al., 2014).

## 3 Results

### 3.1 Composition of gut microbiome at different altitudes

Using these 22 samples, we identified 36036 OTUs based on 98.65% nucleotide sequence identification total reads. A total of 1460 OTUs were shared among samples from the three populations of *P. axillaris* at different altitudes. The unique OTUs from HA, IA, and LA were 12085, 4040, and 13539, respectively (Supplementary Appendix Figure 1A).

### 3.2 The community structure of the gut microbiome at different altitudes

**FIGURE 1:**
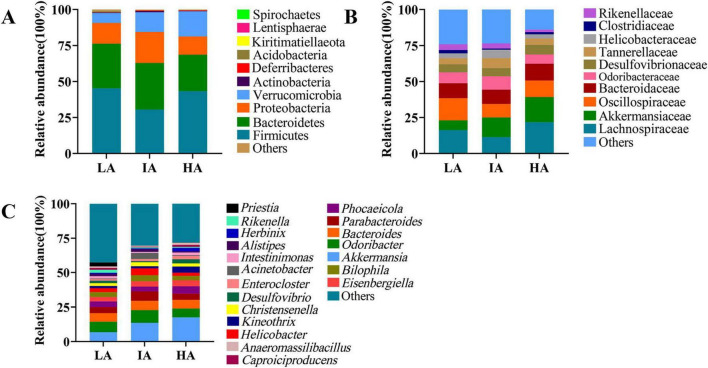
The relative abundance of the gut microbiome at the phylum **(A)**, family **(B)**, and genus **(C)** levels in three populations of *Phrynocephalus axillaris* at different altitudes. Different colors in the figures indicate the different microbes composition, and details are shown on the right sides of each figure, respectively.

At the phylum level, the dominant microbiota were Firmicutes (42.01 ± 2.01%), Bacteroidetes (29.21 ± 1.83%), Proteobacteria (14.86 ± 1.77%), and Verrucomicrobia (11.98 ± 1.75%). At the family level, the dominant microbiota were Lachnospiraceae (17.47 ± 2.34%), Oscillospiraceae (12.83 ± 1.16%), Akkermansiaceae (11.96 ± 1.75%), Bacteroidaceae (10.70 ± 1.12%). At the genus level, the dominant microbiota were *Akkermansia* (11.96 ± 1.75%), *Odoribacter* (7.58 ± 1.11%), *Bacteroides* (6.25 ± 0.69%), and *Parabacteroides* (4.79 ± 0.70%) (Figure 1).

### 3.3 Alpha diversity of gut microbiome at different altitudes

**FIGURE 2:**
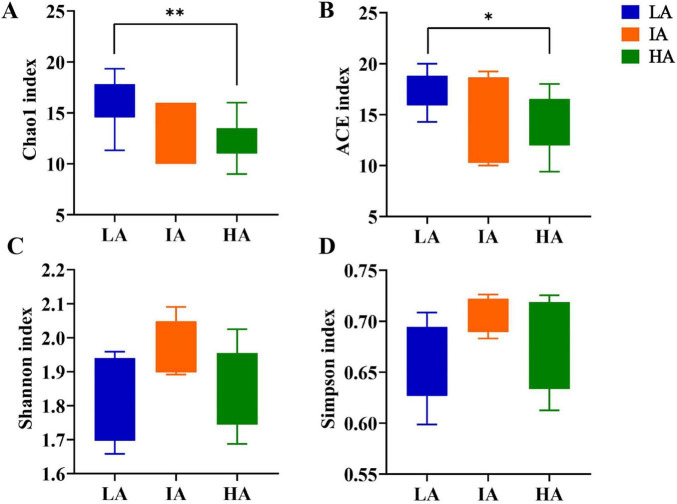
Boxplots of phylum level gut microbiome alpha-diversity of the three populations of *Phrynocephalus axillaris* at different altitudes. **(A)** Chao1 index; **(B)** ACE index; **(C)** Shannon index; **(D)** Simpson index (*P* < 0.05 indicated by*, *P* < 0.01 indicated by**).

Alpha diversity for different altitude groups were compared based on one-way ANOVA test at the phylum level, and the results showed that the community richness (Chao 1 index, ACE index) at LA was higher than that of HA (*P* = 0.009 < 0.01, *P* = 0.042 < 0.05) (Figures 2A, B). The community diversity (Shannon index, Simpson index) was not significantly different (Figures 2C, D), showing a gradual decrease in the abundance of the gut microbiome with increasing altitude.

At the OTU level, principal coordinate analysis (PCoA) based on Bray-Curtis distance found that there was a significant separation among three elevation populations of *P. axillaris*, and the Adonis test also showed that there was a significant difference among them (Adonis test, R^2^ = 0.1474, *P* = 0.001; Supplementary Appendix Figure 1B).

### 3.4 Differences in the gut microbiome composition at different altitudes

**FIGURE 3:**
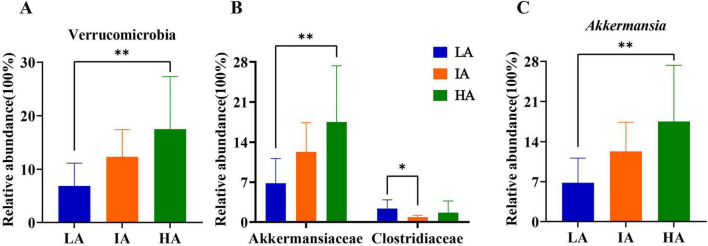
Differences in the relative abundance of microbiome at phyla **(A)**, family **(B)**, and genus **(C)** levels in three populations of *Phrynocephalus axillaris* at different altitudes. *P* < 0.05 indicated by*, *P* < 0.01 indicated by**.

Based on Kruskal-Wallis H, at the phylum level, the relative abundance of Verrucomicrobia differed significantly among the three populations, HA was higher than LA (*P* = 0.005 < 0.01). Further, at the family level, compared with LA, the relative abundance of Akkermansiaceae was significantly increased (*P* = 0.005 < 0.01) at HA. Compared with LA, the relative abundance of Clostriiaceae was significantly decreased (*P* = 0.005 < 0.01) at IA. At the genus level, compared with LA, the relative abundance of *Akkermansia* was significantly increased (*P* = 0.005 < 0.01) at HA (*P* = 0.005 < 0.01) (Figure 3). Among them, the relative abundance of Verrucomicrobia, Akkermansia, and *Akkermansia* increased with altitude.

**FIGURE 4:**
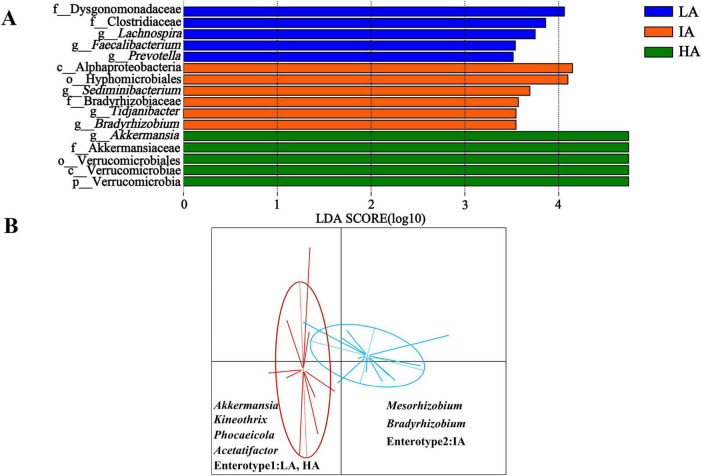
Differences in gut microbiome taxonomic composition among three elevation populations of *Phrynocephalus axillaris* at different altitudes. **(A)** Differences in gut microbiome determined by linear discriminate analysis of effect size (LEfSe) among three populations of *P. axillaris* at different altitudes. **(B)** Gut microbiome enterotype associated with elevation using Bray-Curtis dissimilarity of *P. axillaris*. The highlighted taxa were significantly enriched in the group that corresponds to each color (*P* < 0.05). Linear discriminatory analysis (LDA) scores >3.5.

LEfSe analysis shows that the abundance of f_Dysgonomonadaceae and f_Clostridiaceae were higher enrichment at LA, Akkermansiaceae was higher enrichment at HA (Figure 4A).

Two distinct enterotypes were formed from 22 samples. Each enterotype was driven by the variation of its representative genera level: *Akkermansia*, *Kineothrix*, and *Phocaeicola* in enterotype 1, occurring at LA and HA. *Mesorhizobium* and *Bradyrhizobium* in enterotype 2, occurring at IA (Figure 4B).

### 3.5 Functional prediction of gut microbiome at different altitudes

**FIGURE 5:**
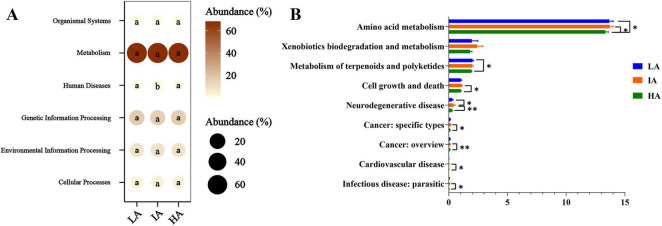
Different functions of gut microbiome of three populations of *Phrynocephalus axillaris* at different altitudes. The microbial functions were predicted using PICRUSt2 at the first **(A)** and the second **(B)** level of the KEGG pathway and were expressed as relative abundances. *P* < 0.05 indicated by*, *P* < 0.01 indicated by**.

KEGG analysis showed that the predicted functions in gut microbiota were mainly involved in Metabolism (67.98% ± 0.23%), Genetic Information Processing (16.35% ± 0.20%), Environmental Information Processing (9.33% ± 0.20%), Cellular Processes (4.08% ± 0.09%), Organismal Systems (1.19% ± 0.01%), and Human Diseases (1.09% ± 0.03%) at the first level. Based on one-way ANOVA tests, significant differences in the metabolic pathways of Human Diseases among the three populations (Figure 5A). In the differential analysis of the KEGG metabolic pathways at the second level, IA showed significantly higher than HA such as Amino acid metabolism, Cell growth and death, Neurodegenerative disease, Cancer:specific types, Cancer: overview, Cardiovascular disease, and Infectious disease: parasitic. The metabolic pathways of Amino acid metabolism, Metabolism of terpenoids and polyketides of LA were significantly higher than HA (Figure 5B).

### 3.6 Fecal metabolic analysis and screening

**FIGURE 6:**
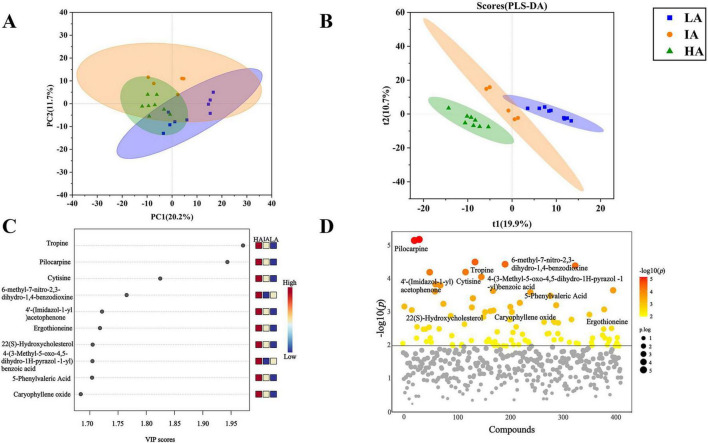
Principal coordinate analysis (PCA) **(A)** and PLS-DA analysis diagram **(B)** based on metabolomics of the three populations of *Phrynocephalus axillaris* at different altitudes. Metabolites variable importance of projection (VIP) analysis **(C)** and ANOVA test of metabolites (VIP > 1) **(D)** in fecal sample from three populations of *P. axillaris* at different altitudes, metabolites above the solid line had significant differences.

There were 1649 metabolites obtained by LC-MS metabolic analysis among three populations of *P. axillaris* at different altitudes. The PCA score plot showed significant differences in metabolites at different altitudes (PC1: 20.2%, PC2: 11.7%) (Figure 6A). The PLS-DA model was efficient and reliable with a low risk of overfitting (Q2 = 0.88, R2 = 0.97, *P* = 0.03 < 0.05). The metabolic curves were clearly different at different altitudes, which indicates obvious differences in fecal metabolites of three populations of *P. axillaris* at different altitudes (Figure 6B). VIP analysis of fecal metabolites from three populations identified the top 10 key metabolites (Figure 6C). The five metabolites, including Tropine, Pilocarpine, 4′-(Imidazol-1-yl)acetophenone, Ergothioneine, and 5-Phenylvaleric Acid tend to be higher with increasing altitude (Figure 6D).

The OPLS-DA analysis was performed on the pairwise metabolite data with par scaling, consisting of HA, IA, and LA (Supplementary Appendix Figure 2). Between the two sample groups of LA and IA, LA and HA, and IA and HA (Q2 = 0.788, R2Y = 0.99; Q2 = 0.779, R2Y = 0.999; Q2 = 0.747, R2Y = 0.99), indicated an excellent model and the metabolic curves were clearly different. Differential metabolites were screened based on the criteria of log_2_FC ≥ 1, OPLS-DA_VIP ≥ 1, *P* ≤ 0.05 to get the top 5 metabolites in each group (Supplementary Appendix Figure 3). Compared with IA, the level of L-arginine, 3-Methyl-2-oxobutanoic acid showed significant decrease at LA, which they were mainly involved in the metabolism of amino acids.

The absolute values of log2FC (Fold Change, FC) were sorted to get the top 10 metabolites in each group (Supplementary Appendix Figure 4). As compared to IA, Prostaglandin D3, 19(R)-Hydroxy-prostaglandin E2, Cannabigerolic acid, isoleucine, 5-Methyluridine were found to be significantly higher at LA. As compared to HA, the metabolites of Glycolithocholic acid and N-Acetyl-L-methionine increased significantly at IA.

## 4 Discussion

Understanding how ectotherms respond and eventually adapt to different altitudes is of great relevance in evolutionary biology, not only to better interpret how they thrive under conditions of increasing environmental harshness but also to predict how endangered species can respond to rapid climate change (Serén et al., 2023). The gut microbiome is highly relevant as it might be related to ecophysiological adaptations of ectotherms to environmental changes in the context of elevation change (Bestion et al., 2017). *P. axillaris* is distributed from high altitude mountain environments to the low altitudual Turpan Basin due to its exceptional wide-ranging adaptations (Arzigul, 2021). Their unique environments are characterized by harsh climates, with high altitude being characterized by low temperatures, low oxygen, strong UV, and low altitude being characterized by high temperatures and drought. Altitudinal changes affect the diversity, structure, and stability of the gut microbiome, which has evolved unique physiological adaptations to different environments (Du et al., 2022). The gut microbiome of the three populations of *P. axillaris* at different altitudes was significantly distinct in terms of diversity, composition, and function, reflecting the plasticity of the gut microbiome that facilitates adaptation to different altitudes.

### 4.1 Dominant gut microbiome at different altitudes

The dominant gut microbiome remained relatively stable despite changes with different elevations. The dominan gut microbiome of *P. axillaris* was dominated by Firmicutes and Bacteroidetes, consistent with previous descriptions of vertebrate (including lizard) (Ibáñez et al., 2021; Youngblut et al., 2019), demonstrating the functional importance of microbiome and ecological significance. Firmicutes and Bacteroidetes are involved in the metabolism of carbohydrates, short-chain fatty acids, and vitamins (Lapébie et al., 2019; Lara et al., 2017), and play an important role in the regulation of host metabolism, and the maintenance of gut microbiome stability. The higher abundance of Lachnospiraceae and Oscillospiraceae, which are abundant in the gut of mammals, particularly humans and ruminants, has been shown to be associated with the production of butyrate, which has an important role in the maintenance of colonic epithelial tissue (Duncan et al., 2002; Zhang et al., 2019). In addition, Lachnospiraceae are involved in carbohydrate metabolism, fatty acid synthesis and degradation, branched-chain amino acid biosynthesis, purine and urea metabolism, and folate biosynthesis (Lin et al., 2024). The abundance of *Oscillospira* increased in response to prolonged fasting in lizards and other vertebrates as well, probably promoting the degradation of some glycans of the host (Buglione et al., 2022). Wildlife are more likely to face unpredictable environments, such as food or water shortages, rapid changes in temperature, etc., and the nutrient compensation of gut microbiome under adverse conditions is crucial for animal survival.

### 4.2 Potential adaptation of the gut microbiome to high altitude

The Chao1 and ACE richness index of the gut microbiome of *P. axillaris* decreased with increasing altitude. The decrease in Alpha diversity of gut microbiome in the high altitude population of *P. vlangalii* was considered to be an important manifestation of hypoxic acclimatization (Zhang et al., 2018). It is worth noting that the elevation range of *P. vlangalii* was 2900–4250 m, all of which were in the high-elevation interval, whereas *P. axillaris* covered the range of very low to intermediate-high elevations.

The relative abundance of Verrucomicrobia, which is the fourth most abundant gut microbiome in *P. axillaris*, increased gradually with elevation and was enriched in HA. This was caused by the increase in the relative abundance of Akkermansiaceae, *Akkermansia*. Akkermansiaceae are mucus-degrading bacteria living in the mucus layer of the gut (Belzer and De Vos, 2012), and their ability to adhere to the mucus layer is considered to be a typical characteristic of probiotics. It has been found that Akkermansiaceae may be one of the immunomodulators that are negatively associated with human diseases such as obesity, diabetes, inflammation, and metabolic disorders (Roopchand et al., 2015; Zhang et al., 2019), and potential bio-indicators for cancer patients (Huang et al., 2021; Kim et al., 2021). It has been found that Akkermansiaceae (*Akkermansia muciniphila*) can adhere to the gut mucosal interface between the lumen and host cells to protect epithelial cells from pathogenic microbes. Potential probiotics enriched at high altitude are likely to help *P. axillaris* improve their metabolic capacity and maintain host health to adapt to the harsh, cold, and hypoxic high altitude environment, where they are highly competitive on a restricted diet of low calorie and nutrient levels at high altitude (Derrien et al., 2004).

The results of PICRUSt2 showed significant differences in metabolic pathways such as Amino acid metabolism, and the relative abundance at IA was significantly higher than at HA, which suggests that high-altitude environments alter the gut microbiome, leading to changes in physiological and metabolic functions. It has been shown that the low-temperature, low-oxygen environment at high altitude threatens biological function and survival, and the body’s self-protection produces an adaptive response to reduce energy demand (Murray and Montgomery, 2014). Metabolically, Glycolithocholic acid and N-Acetyl-L-methionine were significantly decreased in the HA compared to IA. Glycolithocholic acid is a secondary bile acid metabolite that plays an important role in the host and in disease (Cai et al., 2024), and N-Acetyl-L-methionine has antioxidant effects on fats and proteins (Liu Y. et al., 2024). It has been shown that the major oxidative factors UVR, temperature, and oxygen pressure decrease with altitude, resulting in a less oxidative environment at high altitude (Costantini, 2014), and therefore less oxidative damage in high-altitude lizard populations (Reguera et al., 2015), with down-regulation of the relevant metabolites involved.

### 4.3 Potential adaptations of the gut microbiome to low altitude

LEfSe analysis showed that f_Dysgonomonadaceae, f_Clostridiaceae, g_*Lachnospira*, g_*Faecalibacterium*, and g_*Prevotella* appeared to be enriched in *P. axillaris* at LA. Among them, f_Dysgonomonadaceae and f_Clostridiaceae are involved in the digestion and degradation of polysaccharides in food as thermo-tolerant bacteria (Appert et al., 2020; Jiang et al., 2017; Murakami et al., 2018). g_*Lachnospira*, g_ *Faecalibacterium* are associated with intestinal diseases (Vacca et al., 2020). Organisms inhabiting extreme environments have evolved extreme environmental adaptations in order to avoid extinction (Berihulay et al., 2019). The enrichment of the gut microbiome at low altitude Turpan Basin with the extremely dry and hot climate, reveals the effect of heat stress on the intestinal mucosal barrier (Koch et al., 2024; Wen et al., 2021).

L-arginine and 3-methyl-2-oxobutanoic acid are involved in the absorption and utilization of proteins high-regulated in IA, compared with LA (Hallen et al., 2013). Prostaglandin D3 and 19(R)-Hydroxy-prostaglandin E2, which are involved in lipid metabolism (Sam et al., 2012), are high-regulated in LA, compared with IA. A related study demonstrated that the intestinal symbiotic bacterium *Klebsiella michiganensis* modulates L-arginine levels, thereby enhancing resistance to low-temperature stress in the orange fruit fly (*Bactrocera dorsalis*) (Raza et al., 2020). The intermediate elevations *P. axillaris* appeared to have followed a similar metabolic pattern over a long evolutionary period. Temperatures can alter the digestive energy of lizards and differentially affect the utilization of proteins and lipids in the insect-dominated diet (Belhadj Slimen et al., 2016; Plasman et al., 2019), with elevated temperatures increasing the lipid metabolism of lizards (Schramm et al., 2018; Wang et al., 2019), and protein synthesis, and degradation metabolism decreases (Ríus, 2019). The series of metabolite changes may be due to the effects of the extremely high environmental temperatures in the Turpan Basin.

### 4.4 Similar response of gut microbiome to different stresses at low and high altitude

A study of *Sceloporus grammicus* across different altitudes revealed no significant correlation between gut microbiome diversity and elevation (Montoya-Ciriaco et al., 2020). The gut microbiome shaping was associated with changes in diet, met-barcoding based dietary studies of *P. axillaris* found no significant differences in invertebrate diversity in the diets of the LA, IA, and HA (unpublished data), which seems to indicate that there is no a strong correlation between the shaping of gut microbiome and diet in this species, a pattern that is also frequently observed in studies on mammals (Guo et al., 2021). This pattern places greater emphasis on the nutritional convergence of gut microbiome with food, thereby increasing ecological competitiveness and limiting colonization by exogenous microbes (Hernandez-Baixauli et al., 2024).

Gastrointestinal parasites affect the immune system and health of the host (Barelli et al., 2019). However, host-parasite interactions are complex, and the gut microbiome may be a source of parasitic infection (Gouba and Drancourt, 2015). A met-barcoding based study of the parasites of *P. axillaris* found no significant differences in parasite abundance in the gut among the LA, IA, and HA (unpublished data), suggesting that the pattern of altitudinal changes in parasites may be complex and related to the lizard’s own tolerance and other natural environmental factors.

Lizard populations are exposed to extreme heat and cold for longer periods of time in the LA and HA, which promotes prolonged periods of inactivity and limits foraging time of lizards, leading to nutritional stress (Zamora-Camacho et al., 2013). Additionally, higher altitude populations grow slower, store less lipids, are smaller, produce fewer offspring (Hodkinson, 2005), and will have reduced fecundity, while at lower altitudes molecular pathways associated with injury will be upregulated due to high temperatures (e.g., cellular stress response), leading to increased population mortality (Schaefer, 2014) and reduced survival. These gradient changes in fecundity and survival combine to result in a sharp decline in the fitness of populations at low and high elevations, while peak fecundity and survival are reached at intermediate elevations (Buckley et al., 2021; Serén et al., 2023). On the other hand, organisms will evolve strategies to adapt to low and high altitude environments, where metabolic enzymes will be selected to up-regulate energy production rates in response to shorter growing seasons (Marden, 2013; Seebacher, 2018) and to enhance fecundity at high altitudes, and at low altitudes, where extreme environmental conditions (e.g., heatwaves) will be mediated through mechanisms such as heat-shock protein expression and anti-oxidative stress resistance leading to animal selection for enhanced heat tolerance (MacMillan, 2019).

The gut microbiome regulates host responses to the external environment, and interactions between host and gut microbiome influence animal physiological performance and adaptations. The gut microbiome of *P. axillaris* produces similar adaptations to different stressful environments at low and high altitudes, forming 2 different enterotypes, enterotype 1 is found in LA and HA, with representative genera *Akkermansia*, *Kineothrix*, and *Phocaeicola*, and enterotype 2 is found in IA, with representative genera *Mesorhizobium*, *Bradyrhizobium*. Enterotype 1 is associated with immune stress. *Akkermansia* metabolizes nutrients to provide energy for growth (Chelakkot et al., 2018; Hänninen et al., 2018) and can degrade gut mucin to produce short-chain fatty acids such as acetate, propionate, etc. *Kineothri*x ferments sugar to produce butyrate (Haas and Blanchard, 2017). *Phocaeicola* can degrade complex polysaccharides into short-chain fatty acids that are involved in the synthesis of vitamins and other bioactive compounds (Lück and Deppenmeier, 2022). Short-chain fatty acid production maintains gut immune status and plays an important role in metabolism, inflammation, and disease (Feng et al., 2018; Rooks and Garrett, 2016; Shealy et al., 2021). *Mesorhizobium* and *Bradyrhizobium* in enterotype 2, are not typical in the animal gut, but are considered environmentally beneficial microorganisms and are mainly found in soil (Favero et al., 2021; Laranjo et al., 2014). *Mesorhizobium* produces rhizobial proteins that provide metabolic and stress-related pathways for plants (Diouf et al., 2015), and associate host interactions and disease progression as an environmentally carrying symbiotic bacterium (Ebenezer et al., 2019). *Bradyrhizobium* also has the ability to maintain general cellular functions, such as amino acid transport and metabolism, energy production and conversion (Zhong et al., 2024), shows strong positive correlations with another gut microbiome in insects and plays an important role in cold and hot environments (Mazzucco and Schlötterer, 2021). However, the role of enterotype2 dominated by *Mesorhizobium* and *Bradyrhizobium* in animal gut is understudied, and should be requires further investigation.

Altitude changes are inevitably accompanied by changes in a variety of environmental factors, such as, temperature, humidity, oxygen content, etc., and the effects of altitude change on organisms are complex (Liu D. et al., 2024). In a study of *Sceloporus grammicus*, it was shown that the abundance of *Akkermansia* (Verrucomicrobia) increased significantly at high altitudes with low oxygen partial pressure and low temperatures (Montoya-Ciriaco et al., 2020). However, this change may not be directly due to altitude, but rather to variations in food composition in different altitudinal contexts. Recently studies have focused on how high-altitude environments with low oxygen levels, low temperatures, and intense radiation affect animal reproduction and survival, influencing not only species evolution and physiological adaptation but also the composition of animal gut microbiota (Bai et al., 2022; Wang et al., 2022). Therefore, multiple variable factors in a complex altitudinal context can jointly determine animal gut microbes. The shaping of gut microorganisms in *P. axillaris* by changes in altitude should also be a joint effect of multiple environmental factors. In the future, the degree of contribution of a particular factor to the formation of gut microbiome in this species should be evaluated under the premise of strictly controlling variation environmental factors.

## 5 Conclusion

Our study shows that *P. axillaris* has significant differences in gut microbiome composition, abundance, functional pathways, and metabolites among populations at different altitudes. Such differences may be an adaptation to stressors at different altitudes. The dominant gut microbiome of Firmicutes, Bacteroidetes, Lachnospiraceae, and Oscillospiraceae remained relatively stable across different altitudes, this stable may be a key factor in the strong adaptive ability of *P. axillaris. Akkermansia* was enriched in high-altitude populations likely in response to low temperature and hypoxic conditions. The significant enrichment of heat-resistant microbiome in low-altitude populations may be an adaptation to high temperature. The plasticity of specific microbiome in the gut may be the main way for the species to flexibly respond to environmental changes. Under similar ecological stresses, low and high-altitude populations shared an enterotype dominated by *Akkermansia*, *Kineothrix*, *Phocaeicola.* Additionally, most metabolite profiles demonstrated the adaptation of gut microbiota to environments at different altitudes.

In the face of rising global temperatures, lizards have limited foraging time, morphological, physiological and behavioral functions may be sub-optimal. On the one hand, *P. axillaris*, as a widely distributed species, with the stability of its dominant microbiome and the plasticity of its signature taxa, is highly adaptable to cope with changes in low and high altitude environments. On the other hand, with global warming, the species approach a heat-tolerance threshold at low altitude, leading to a decline in biological performance and adaptability. In conclusion, understanding the adaptability of wide-ranging species to different environments can help to respond to global warming.

## Data Availability

The datasets presented in this study can be found in online repositories. The names of the repository/repositories and accession number(s) can be found below: https://www.ncbi.nlm.nih.gov/, PRJNA1160045.

## Footnotes

1
http://reptile-database.org/

2
http://drive5.com/uparse/

3
https://ngdc.cncb.ac.cn/databasecommons/database/id/237

4
https://github.com/picrust/picrust2

5
https://www.mzcloud.org/

6
https://www.genome.jp/kegg/pathway.html

## Supplementary material

Supplementary Appendix Figure 1 Differences in gut microbiome taxonomic composition of three populations of *Phrynocephalus axillaris* at different altitudes. **(A)** Venn diagram showing the unique and shared OTUs levels among three elevation populations of *P. axillaris.* **(B)** Principal coordinate analysis (PCoA) plot of the OTUs level gut microbiome Beta-diversity of the three elevation populations of *P. axillaris.*

Supplementary Appendix Figure 2 OPLS-D analysis of the metabolic samples of three populations of *Phrynocephalus axillaris* at different altitudes. **(A)** LA vs IA, **(B)** LA vs HA, **(C)** IA vs HA.

Supplementary Appendix Figure 3 Annotation and screening of metabolites of three populations of *Phrynocephalus axillaris* at different altitudes. **(A)** LA vs IA, **(B)** LA vs HA, **(C)** IA vs HA. The volcanic map was used for statistical analysis of differential metabolites in fecal, and the horizontal coordinate indicated the quantitative value of differential metabolites to calculate the corresponding ratio. The ordinate represents the *P*-value of the metabolite in the *T*-test between the groups. The volcano plot of the metabolites, red points represent up-regulated metabolites, green point represented down-regulated metabolites and gray points represent have no significant differences metabolites. The top 5 differential metabolites were selected and labeled.

Supplementary Appendix Figure 4 The bidirectional bar chart was used for statistical analysis of the multiple changes of fecal differential metabolites of three populations of *Phrynocephalus axillaris* at different altitudes, with the horizontal coordinate representing log2 (FC) and the vertical coordinate representing the names of the top ten differential metabolites in different altitude groups. **(A)** LA vs IA, **(B)** LA vs HA, **(C)** IA vs HA. Dark blue represented down-regulated metabolites and light blue represent up-regulated metabolites.
