# Supplementary material for: All roads lead to Rome: the plasticity of gut microbiome drives the extensive adaptation of the Yarkand toad-headed agama (Phrynocephalus axillaris) to different altitudes
Source: Front Microbiol. 2025 Jan 8;15:1501684. doi: 10.3389/fmicb.2024.1501684 (PMC11751238; doi:10.3389/fmicb.2024.1501684)
Supplement: Supplementary file 1 [file Table_1.docx]

Appendix Table 1 Environmental parameters of three populations of *Phrynocephalus axillaris* at different altitudes(including Annual Mean Temperature, Annual Mean Precipitation).

| Name | Annual Mean Temperature（℃） | Annual Mean Precipitation（mm） | Precipitation Seasonality（mm） |
| --- | --- | --- | --- |
| LA-1 | 14.95 | 29.00 | 47.45 |
| LA-2 | 14.95 | 29.00 | 47.45 |
| LA-3 | 14.95 | 29.00 | 47.45 |
| LA-4 | 14.95 | 29.00 | 47.45 |
| LA-5 | 14.95 | 29.00 | 47.45 |
| LA-6 | 14.95 | 29.00 | 47.45 |
| LA-7 | 15.15 | 15.00 | 63.21 |
| LA-8 | 14.44 | 28.00 | 50.09 |
| LA-10 | 11.97 | 37.00 | 59.49 |
| IA-1 | 9.87 | 110.00 | 68.25 |
| IA-2 | 11.88 | 42.00 | 96.40 |
| IA-3 | 11.88 | 42.00 | 96.40 |
| IA-4 | 8.88 | 117.00 | 70.97 |
| IA-5 | 8.88 | 117.00 | 70.97 |
| HA-1 | 8.17 | 45.00 | 102.01 |
| HA-2 | 8.17 | 45.00 | 102.01 |
| HA-3 | 8.17 | 45.00 | 102.01 |
| HA-4 | 8.17 | 45.00 | 102.01 |
| HA-5 | 8.17 | 45.00 | 102.01 |
| HA-6 | 8.17 | 45.00 | 102.01 |
| HA-7 | 8.17 | 45.00 | 102.01 |
| HA-8 | 8.17 | 45.00 | 102.01 |
