# Supplementary figures and images for: All roads lead to Rome: the plasticity of gut microbiome drives the extensive adaptation of the Yarkand toad-headed agama (Phrynocephalus axillaris) to different altitudes
Source: Front Microbiol. 2025 Jan 8;15:1501684. doi: 10.3389/fmicb.2024.1501684 (PMC11751238; doi:10.3389/fmicb.2024.1501684)

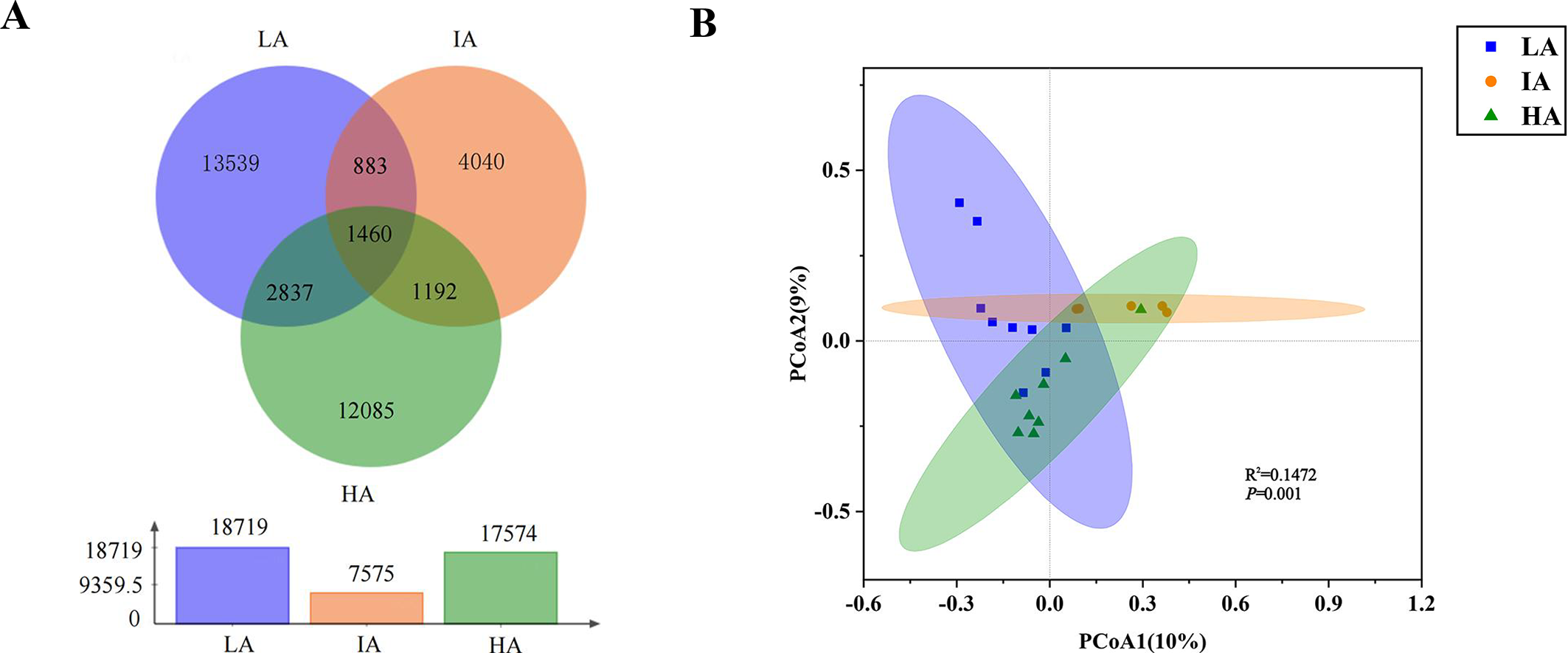

Supplement: Supplementary Appendix Figure 1 — Differences in gut microbiome taxonomic composition of three populations of Phrynocephalus axillaris at different altitudes. (A) Venn diagram showing the unique and shared OTUs levels among three elevation populations of P. axillaris. (B) Principal coordinate analysis (PCoA) plot of the OTUs level gut microbiome Beta-diversity of the three elevation populations of P. axillaris. [file Image_1.tif]

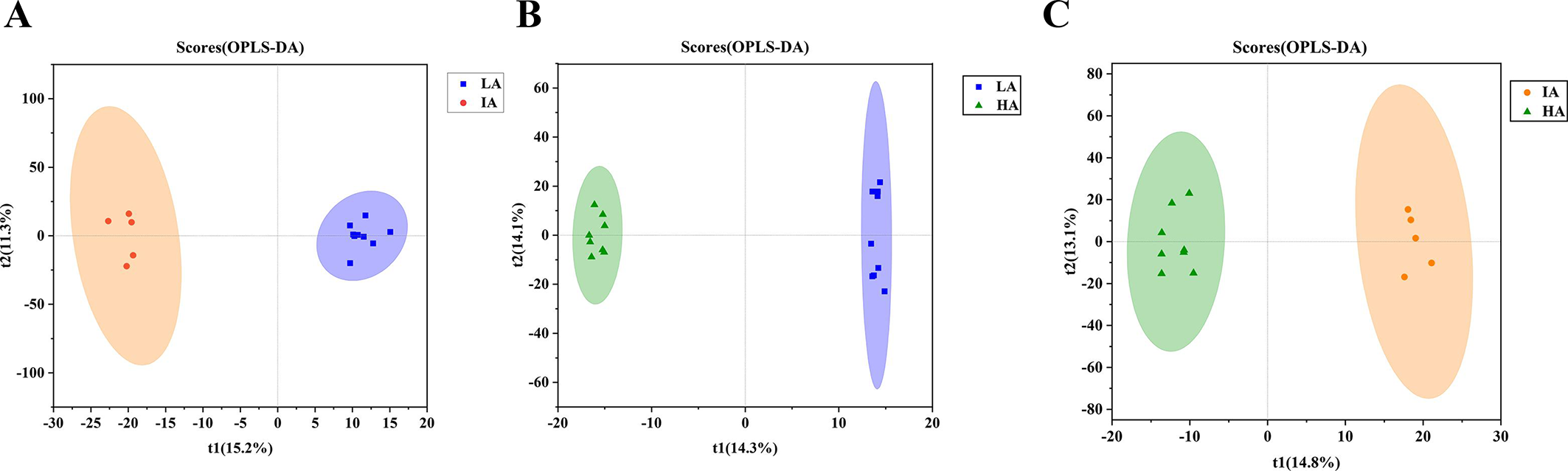

Supplement: Supplementary Appendix Figure 2 — OPLS-D analysis of the metabolic samples of three populations of Phrynocephalus axillaris at different altitudes. (A) LA vs IA, (B) LA vs HA, (C) IA vs HA. [file Image_2.tif]

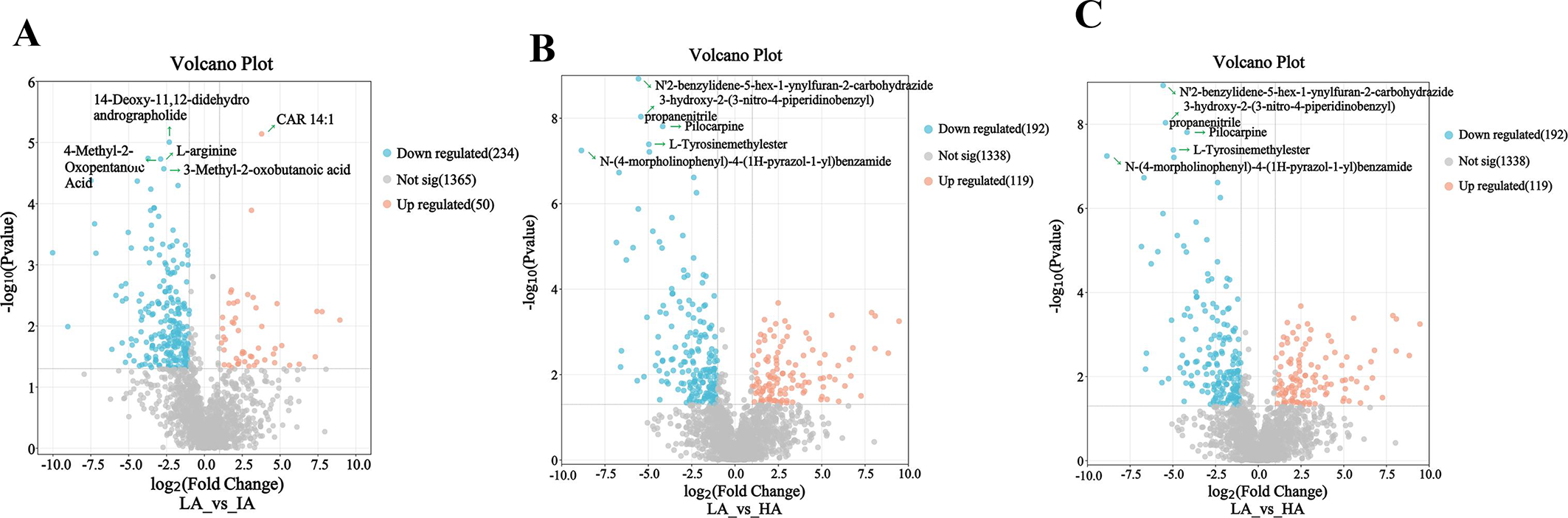

Supplement: Supplementary Appendix Figure 3 — Annotation and screening of metabolites of three populations of Phrynocephalus axillaris at different altitudes. (A) LA vs IA, (B) LA vs HA, (C) IA vs HA. The volcanic map was used for statistical analysis of differential metabolites in fecal, and the horizontal coordinate indicated the quantitative value of differential metabolites to calculate the corresponding ratio. The ordinate represents the P-value of the metabolite in the T-test between the groups. The volcano plot of the metabolites, red points represent up-regulated metabolites, green point represented down-regulated metabolites and gray points represent have no significant differences metabolites. The top 5 differential metabolites were selected and labeled. [file Image_3.tif]

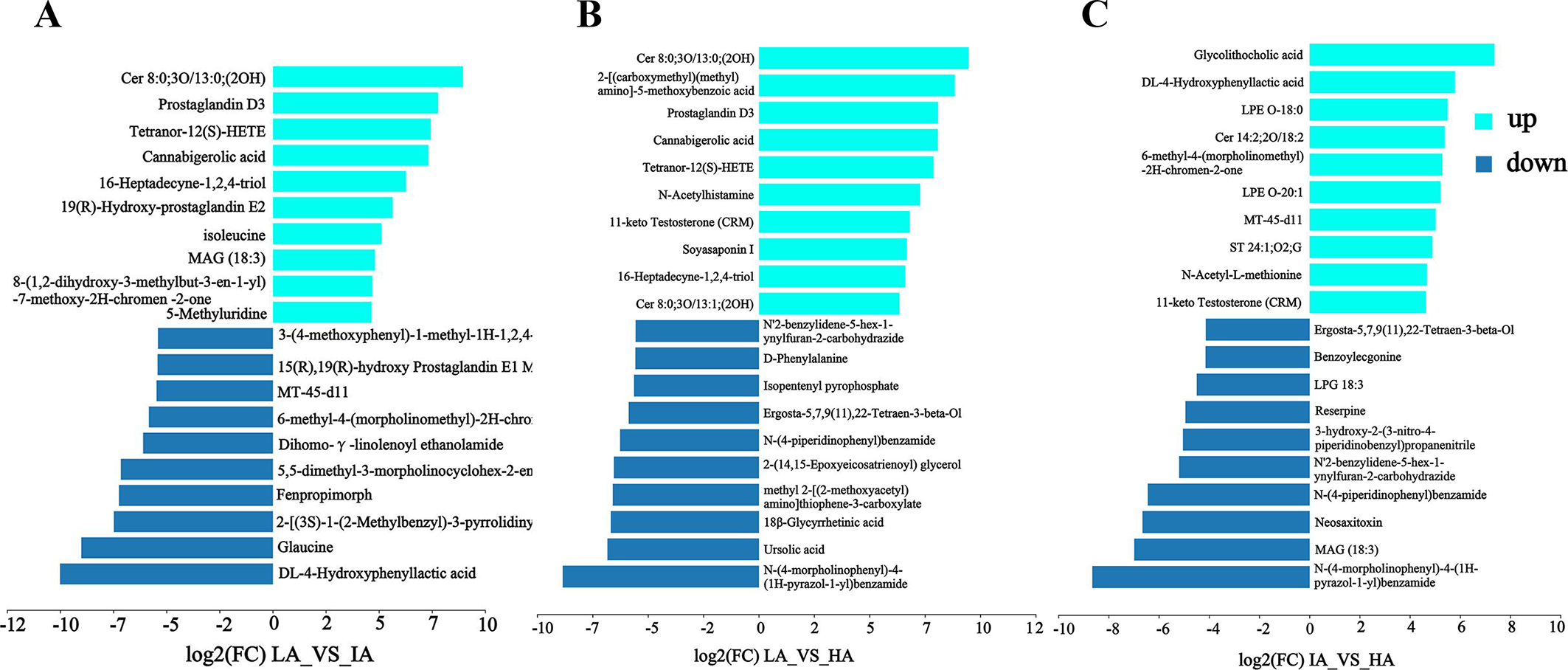

Supplement: Supplementary Appendix Figure 4 — The bidirectional bar chart was used for statistical analysis of the multiple changes of fecal differential metabolites of three populations of Phrynocephalus axillaris at different altitudes, with the horizontal coordinate representing log2 (FC) and the vertical coordinate representing the names of the top ten differential metabolites in different altitude groups. (A) LA vs IA, (B) LA vs HA, (C) IA vs HA. Dark blue represented down-regulated metabolites and light blue represent up-regulated metabolites. [file Image_4.tif]
